# Menopausal Hormone Therapy—Risks, Benefits and Emerging Options: A Narrative Review

**DOI:** 10.3390/ijms262211098

**Published:** 2025-11-17

**Authors:** Ana Maria Arnautu, Vanda Roxana Nimigean, Claudia Alexandra Nacea-Radu, Dana Mihaela Tilici, Diana Loreta Paun

**Affiliations:** 1 Endocrinology Department, Bucharest University Emergency Hospital, 050098 Bucharest, Romania; ana-maria.arnautu@rez.umfcd.ro (A.M.A.); dana-mihaela.tilici@drd.umfcd.ro (D.M.T.); diana.paun@umfcd.ro (D.L.P.); 2 Department of Oral Rehabilitation, Faculty of Dentistry, University of Medicine and Pharmacy “Carol Davila”, 020021 Bucharest, Romania; 3 Doctoral School, University of Medicine and Pharmacy “Carol Davila”, 020021 Bucharest, Romania; 4 Faculty of Medicine, University of Medicine and Pharmacy “Carol Davila”, 020021 Bucharest, Romania

**Keywords:** hormone replacement therapy, menopausal hormone therapy, personalized regimens, estetrol

## Abstract

This study aims to synthesize contemporary evidence on the benefits, risks, and emerging options in menopausal hormone therapy (MHT), emphasizing the recent literature. A narrative review of peer-reviewed studies and guidelines (up to September 2025) from major databases (e.g., MEDLINE/PubMed, Embase, Cochrane) was conducted, with emphasis on the last five years and qualitative synthesis. MHT provides the most effective relief of vasomotor symptoms and is the first-line treatment for genitourinary syndrome of menopause, particularly with the use of low-dose local vaginal estrogen preparations; it also prevents early postmenopausal bone loss and reduces fractures in selected cases. Cardiovascular prevention is not an indication. Benefit–risk depends on timing, route, and dose. Initiation within 10 years of menopause as well as the use of transdermal estradiol at low–moderate doses are favored when cardiometabolic or thrombotic risk is salient. In contrast, oral regimens—particularly those using conjugated equine estrogens—are associated with higher risks of venous thromboembolism and stroke compared with transdermal 17 β-estradiol, and risk also varies by the type of progestogen used. Effects on breast cancer risks are regimen-specific: neutral to favorable with estrogen-alone after hysterectomy, but increasing with longer use of combined therapy. While the absolute risk of ovarian cancer remains small, evidence for colorectal cancer remains mixed. MHT confers modest improvements in sleep, mood, intercourse, and quality of life. Estetrol (E4) shows anti-VMS efficacy at the minimum effective oral dose and favorable pharmacology, but conclusive data on its long-term cardiovascular, thrombotic, and breast safety are pending. MHT should be individualized appropriately based on the patient, timing, route, dose, and choice of progestogen. The lowest effective dose should be used, alongside periodically reassessing the therapy as new evidence, including emerging data on E4, emerges.

## 1. Introduction

Menopausal hormone therapy (MHT) is the most effective treatment for moderate-to-severe vasomotor symptoms and, in appropriately selected women, also treats genitourinary syndrome of menopause and prevents postmenopausal bone loss and fractures [1]. Before the early 2000s, MHT was widely prescribed—including for presumed cardiovascular protection—but prescribing dropped sharply after the initial Women’s Health Initiative (WHI) reports, which highlighted safety concerns [2].

Subsequent re-analyses and complementary trials have reframed risk–benefit by age at initiation, time since menopause, route, dose, and progestogen choice. In particular, studies such as ELITE and KEEPS suggested a more favorable vascular profile when therapy is initiated closer to the menopause transition (“timing hypothesis”), while confirming symptomatic efficacy [3,4]. Contemporary syntheses and secondary WHI analyses further support individualized selection of regimen and route rather than a one-size-fits-all approach to prescribing [5].

In parallel, professional societies have updated guidance. Current statements endorse MHT for symptomatic women < 60 years or within 10 years of menopause who lack contraindications (e.g., breast cancer, active thromboembolic disease, uncontrolled cardiovascular disease), emphasizing shared decision-making, the lowest effective dose, and periodic re-evaluation [6].

We present a narrative review assessing the benefits and risks of MHT across key outcomes and examining how initiation timing, formulation, route, and dosage influence the risk–benefit profile to inform patient-centered decisions. Additionally, we present recent information about estetrol, which is under evaluation for menopausal hormone therapy.

This narrative review summarizes contemporary evidence on MHT, drawing from peer-reviewed literature published up to September 2025. Relevant studies were identified through searches of MEDLINE/PubMed, Embase, Cochrane Library, Web of Science, and Scopus databases, complemented by clinical guidelines and position statements from major professional societies. Seminal trials such as the Women’s Health Initiative and recent systematic reviews were included to provide context and highlight evolving concepts regarding timing, route, formulation, and emerging agents such as estetrol. Given the heterogeneity of study designs and outcomes, a qualitative synthesis approach was used to integrate key findings and practice implications.

## 2. Literature Overview

### 2.1. Types of MHT

Menopausal hormone therapy replaces declining ovarian hormones across the menopause transition to relieve symptoms—principally vasomotor symptoms—and, in appropriately selected women, to prevent postmenopausal bone loss and fractures. FDA-approved indications include moderate-to-severe VMSs and prevention of osteoporosis [7].

Regimens are tailored to uterine status: estrogen therapy (ET) for women without a uterus and estrogen–progestogen therapy (EPT) for those with an intact uterus to protect the endometrium. Therapeutic options comprise 17β-estradiol (oral micronized; transdermal patch, gel, spray), conjugated estrogens (e.g., CEEs), and synthetic conjugated estrogens; ethinyl estradiol is primarily a contraceptive estrogen and is not standard in contemporary MHT [7].

Dosing ranges from standard to low and ultra-low, titrated to the lowest effective dose. Routes include oral and transdermal (systemic) and vaginal (low-dose local therapy for GSM), each with distinct pharmacokinetics and selection driven by patient characteristics and preferences [7].

The physiological rationale is the broad impact of estrogen decline: VMSs are common and may persist, while hypoestrogenism promotes central adiposity and insulin resistance, adversely affects endothelial function and lipid profiles, accelerates bone loss (increasing fracture risk), and may be accompanied by transient verbal-memory difficulties during the transition [8]. Prolonged low estrogen status before age 45 is associated with higher long-term risks of cardiovascular disease, osteoporotic fracture, and possibly cognitive impairment [9].

The following table provides a concise reference to estrogen-based MHT options by route, with typical dosing ranges, expected advantages, and adverse effects and guidance for regimen selection in clinical practice (Table 1).

Consistent with risk-minimization principles, menopausal hormone therapy should be initiated at the lowest effective dose, with route and regimen individualized, then titrated to symptom control and tolerability (Table 2).

### 2.2. Benefits of MHT

#### 2.2.1. VMS Control: Efficacy and Onset

Hormone therapy is the most effective treatment for menopause-related vasomotor symptoms, consistently outperforming placebo and nonhormonal options. Across randomized trials, estrogen therapy (with or without progestogen) reduces hot-flash frequency by roughly 70–80% versus baseline and significantly more than placebo; NAMS estimates a 75% reduction in weekly VMS frequency compared with placebo [6]. Placebo responses are substantial (40–60% reductions in some trials) [11], but active estrogen therapy still confers a large incremental benefit. Symptom relief typically begins within 2–4 weeks and is maximal by 8–12 weeks [12].

Landmark trials provide additional context. In the Women’s Health Initiative, the regimens tested—oral CEEs 0.625 mg/day and oral CEEs 0.625 mg/day plus MPA 2.5 mg/day—were effective for VMS control [13]. Recent re-analyses have reframed risk–benefit by age, timing, and regimen; however, the WHI tested only conjugated equine estrogens with medroxyprogesterone acetate, so its findings cannot be generalized to all estrogen or progestogen types. A recent WHI secondary analysis stratified by age and baseline symptom burden found that, among women 50–59 years with moderate–severe VMSs, both CEE alone and CEE plus MPA reduced VMSs without increasing ASCVD risk; caution is advised in women 60–69 years, and initiation is not recommended at ≥70 years due to higher cardiovascular risk [14]. These findings align with current guidance that supports MHT for VMSs in appropriately selected younger postmenopausal women.

Efficacy is maintained with lower systemic doses (lower-dose CEE-based regimens) and across routes (oral, transdermal), provided adequate estrogen exposure is achieved; progestogen is required for endometrial protection in women with a uterus and does not abrogate VMS benefit [15]. Nonhormonal therapies (SSRIs/SNRIs, gabapentin, clonidine, NK3R antagonists) are reasonable alternatives when MHT is contraindicated or declined, but on average achieve more modest VMS reductions than estrogen therapy [16].

The consensus is that MHT remains the standard of care for moderate-to-severe VMSs, with significant, fast, and clinically important results, but treatment should be tailored to age, duration since menopause, risk profile, formulation, and route.

#### 2.2.2. Genitourinary Syndrome of Menopause (GSM): Local vs. Systemic Therapy

The genitourinary syndrome of menopause is the accepted term used to describe the broad spectrum of genitourinary tract symptoms and signs resulting from the loss of endogenous sex steroids that occurs at and after menopause. Approximately 50–70% of postmenopausal women report symptomatic GSM [17]. Estrogen deficiency induces metabolic and trophic changes in the vagina and other genital tissues, leading them to become thinner, drier, and less elastic. Consequently, low estrogen levels cause genital areas to become dry, itchy, and more easily irritated. Reduced blood flow results in decreased secretions, further exacerbating dryness and making sexual intercourse uncomfortable or painful.

For decades, local vaginal estrogen has been the treatment of choice for postmenopausal women with vulvovaginal symptoms alone. Compared with systemic therapy for vasomotor symptoms, local administration allows for a lower estrogen dose. Available formulations include the estradiol-releasing vaginal ring, estrogen-based vaginal creams, pessaries containing estriol, and a slow-release 17β-estradiol tablet [18]. Notably, about one-third of women on systemic hormone replacement therapy continue to experience GSM symptoms and require additional local estrogen therapy [18].

Recent evidence confirms that intravaginal estrogen is more effective than placebo in the management of vaginal atrophy, with comparable efficacy across different formulations (cream, ring, tablets) [18]. More recent data also suggest potential benefits for vaginal tissue quality and a reduction in urinary tract infections, although further standardized trials are needed to confirm these findings [19].

Finally, current guidelines recommend local estrogen administration over systemic MHT when vaginal atrophy is the only indication for treatment [2].

#### 2.2.3. Bone Health with MHT

Estrogen deficiency triggers accelerated bone loss, peaking within the first 2–3 years after the menopausal transition; however, this process can be effectively prevented with menopausal hormone therapy. It is the only anti-osteoporotic therapy that has a proven efficacy regardless of the basal level of risk, even in low-risk women for fracture [20].

During the first 3–6 months of treatment, estrogens rapidly suppress the increased osteoclastic activity typical of early menopause, leading to a marked reduction in bone resorption. Due to the coupling of bone turnover processes, a subsequent decrease in bone formation follows. Within 6 to 12 months, a new stable state of bone remodeling usually forms, and it lasts as long as estrogen medication is administered [20,21].

Most women initiating low-dose estrogen medication benefit from prevention against early postmenopausal bone loss, according to most studies. The use of lower estrogen dosages than those advised a few years ago is currently widespread because it has been demonstrated that these dosages effectively relieve menopausal symptoms and have a better bleeding profile than higher estrogen dosages.

A recent study of 107 postmenopausal women demonstrated that treatment with 0.5 mg of 17β-estradiol plus 0.1 mg of norethisterone acetate reduced both bone resorption and formation markers—an expected outcome of antiresorptive therapy—suggesting a protective effect on bone even with ultra-low-dose MHT [22].

Although the International Menopause Society [23] and the British Menopause Society [24] support the use of MHT as a first-line option for osteoporosis prevention, most international guidelines recommend bisphosphonates or denosumab as initial therapy. The American Association of Clinical Endocrinologists [25], in accordance with FDA guidance, advises estrogen use only to women at high risk of osteoporosis and only when non-estrogen alternatives are unsuitable, emphasizing the lowest effective dose for the shortest duration. Current consensus limits MHT use to women under 60 years of age, within 10 years of menopause, and without major comorbidities [26].

Thus, although MHT is effective in preserving bone mineral density and reducing fracture risk, its role in osteoporosis prevention remains debated, and further research is warranted—particularly regarding regimens with transdermal estrogen and micronized progesterone.

#### 2.2.4. MHT and Colorectal Cancer (CRC)

Since the first reports in the 1980s, numerous studies have linked MHT use with a lower risk of colorectal cancer. In normal colonic tissue, ERβ is central to epithelial homeostasis and mucosal immune function; its activation by estrogens—including estrogenic flavonoids—can engage tumor-suppressive pathways, which may help explain the observed protection. Mechanistically, the protective effect of MHT is thought to be mediated via nuclear estrogen receptors (ERα, ERβ) and the progesterone receptor, leading to enhanced DNA repair, selective activation of pro-apoptotic signaling, downregulation of oncogene expression, tighter control of cell-cycle progression, and epigenetic modulation (shifts in microRNA profiles and DNA methylation) [27].

Evidence on MHT and colorectal cancer remains mixed. In the randomized Women’s Health Initiative, daily oral conjugated equine estrogens 0.625 mg plus medroxyprogesterone acetate 2.5 mg given for approximately 5.6 years showed no long-term reduction in CRC incidence and no reduction in CRC mortality, despite fewer cases during the intervention phase and more advanced stage at diagnosis—findings that do not support a clinically meaningful benefit for the CEE-plus-MPA oral regimen [28].

By contrast, a 2024 meta-analysis of observational cohorts (over 480,000 participants) reported that exposure to any MHT (formulation or route seldom distinguished) was associated with lower CRC-specific mortality and lower all-cause mortality among women with CRC [29]. Complementary cohort data (over 28,000 participants) suggested that ever-use of any MHT conferred larger 30-year absolute risk reductions among women with high polygenic risk than among those at lower genetic risk [30].

Collectively, trials of oral CEEs plus MPA have not demonstrated CRC risk reduction, while observational cohorts using non-specific MHT exposure definitions have observed modest inverse associations, particularly among genetically susceptible subgroups, warranting cautious interpretation given residual confounding and exposure misclassification.

#### 2.2.5. MHT and Cardiovascular Effects

The relationship between postmenopausal hormone therapy and cardiovascular disease (CVD) remains debated.

Contemporary syntheses indicate that MHT favorably influences endothelial function: in a 2024 systematic review and meta-analysis, MHT significantly improved flow-mediated dilation (FMD) versus placebo in postmenopausal women; early initiation (within 10 years of menopause) was associated with lower all-cause mortality and cardiovascular events and with larger FMD gains. By contrast, nitroglycerin-mediated dilation (NMD) did not improve significantly, pooled analyses did not demonstrate reductions in hard cardiovascular outcomes overall; moreover, risks of stroke and venous thromboembolism were higher, particularly with oral therapy [31].

Route, dose, and timing appear to modify vascular and clinical responses. A systematic review including 33 studies (6 RCTs and 27 cohort studies) examined how route of administration, duration, and dose of MHT relate to cardiovascular outcomes. Based largely on observational data, the authors reported a possible cardioprotective effect even with low-dose oral therapy—0.3 mg/day oral conjugated equine estrogens showed an effect similar to the standard 0.625 mg/day dose.

However, no randomized trials demonstrating cardioprotective benefit for primary prevention were identified. The review also noted that vaginal MHT might reduce myocardial infarction and stroke risk, but the evidence is limited and requires further confirmation [32].

In practice, MHT can improve endothelial function and specific cardiometabolic markers, particularly with early initiation, although there is little evidence of consistent cardioprotection in primary prevention. Because oral regimens are linked to an increased risk of venous thromboembolism and stroke, current guidelines recommend a cautious, individualized risk–benefit assessment and, when systemic therapy is required for symptom control, a preference for transdermal estradiol in women with established CVD or elevated baseline risk [33].

The heterogeneity of cardiovascular effects likely reflects therapy characteristics (type, dose, route), vascular estrogen–receptor biology (numbers, variants, sensitivity), menopausal timing (initiation window and duration), hormonal milieu (progestogen, gonadotropins, androgens, SHBG), and baseline cardiovascular health, underscoring the need for patient-specific selection of regimen and route [32].

#### 2.2.6. Potential Additional Benefits of MHT: Sleep, Mood, Sexual Function, Quality of Life

Beyond vasomotor and genitourinary symptoms, accumulating evidence suggests that menopausal hormone therapy may exert modest but clinically relevant benefits on sleep quality, mood regulation, sexual function, and overall quality of life.

Estrogen therapy appears to improve sleep architecture and reduce insomnia severity, partly through stabilization of thermoregulation and neuroendocrine feedback loops. A 2024 Korean study reported significant improvements in the Pittsburgh Sleep Quality Index (PSQI) after 1 and 3 months of MHT (mean PSQI score reduced from 7.8 to 6.1), indicating better global sleep quality [34]. Similarly, systematic reviews indicate that improvements in sleep frequently accompany reductions in vasomotor symptom burden, particularly nocturnal hot flashes and night sweats [35].

Regarding mood, estrogen supplementation—particularly in early postmenopause—has been associated with modest reductions in depressive symptoms, with transdermal estradiol showing the most consistent benefits [36,37]. These effects are generally more pronounced in perimenopausal women than in late postmenopause, likely due to neurosteroid and serotonergic modulation.

Sexual function may also benefit from systemic or local estrogen therapy. The 2025 Cochrane meta-analysis concluded that estrogen therapy alone presumably slightly improves the overall rating for sexual function in women experiencing menopause or in the early stages of postmenopause, particularly in the areas of lubrication, discomfort, and satisfaction [38]. Improvements in sexual well-being are more pronounced when GSM symptoms (vaginal dryness, dyspareunia) are concurrently treated with low-dose vaginal estrogen or combined systemic therapy [39].

Overall, although effect sizes are modest, improvements in sleep, mood, and sexual comfort highlight the broader, holistic benefits of individualized menopausal hormone therapy. These findings reinforce current NAMS, EMAS, and BMS consensus statements, which recognize quality of life as a central consideration in treatment decisions.

### 2.3. Risks of MHT

#### 2.3.1. Cerebrovascular Risk: Stroke Risk and the Role of MHT

Randomized clinical trials, most notably the Women’s Health Initiative, did not demonstrate cardiovascular protection with menopausal hormone therapy; on the contrary, they raised concerns about increased cardiovascular risk, particularly stroke [40,41]. Subsequent extension studies and subgroup analyses suggest that coronary risk is modified by timing: hazard appears higher when MHT is initiated ≥60 years or >10 years after menopause, whereas younger women may fare better, supporting the “window hypothesis” for CHD outcomes [14,42]. By contrast, evidence for stroke has remained inconsistent across studies.

Contemporary observational data illustrate this heterogeneity. In a large NHIS-NSC cohort (over 550,000 postmenopausal women), current MHT use—especially estrogen-only regimens—was associated with a higher risk of ischemic stroke; importantly, risk returned to baseline after discontinuation, indicating no excess with past use [43]. Conversely, a pooled analysis of Swedish cohorts reported that when MHT was initiated within five years of menopause, it was not associated with an increased risk of incident ischemic stroke, regardless of route, type, active ingredient or duration [44].

Route and dose appear pivotal. A recent nationwide cohort focusing specifically on ischemic stroke found that oral estrogen therapy conveyed risk estimates consistent with randomized trials. In contrast, transdermal estrogen was not associated with excess stroke risk, and low-dose vaginal estrogen was associated with a lower risk [45]. Among women with prior ischemic stroke, the use of vaginal estradiol tablets was not linked to higher recurrence rates, suggesting a neutral cerebrovascular profile in secondary prevention settings [46]. Taken together, these data reinforce earlier indications that low-dose vaginal regimens are cerebrovascular neutral, while acknowledging the observational nature of the evidence.

Current syntheses indicate that higher systemic estrogen doses are associated with greater stroke (and VTE) risk across oral and transdermal routes, whereas transdermal estradiol ≤ 50 μg/day, particularly when combined with micronized progesterone, appears to offer a more favorable thrombotic and cerebrovascular profile [32]. In practice, stroke risk with MHT should be minimized by individualized selection of route, dose, and timing, favoring lower-dose transdermal regimens when systemic therapy is needed, and avoiding initiation at older ages or long after menopause when feasible [14,32].

VTE risk differs by estrogen type and route: transdermal estradiol has the lowest risk, oral estradiol is lower than CEE, and ethinyl estradiol (in combined contraceptives) carries the highest risk. Current guidance favors low-dose, transdermal estradiol regimens when feasible.

#### 2.3.2. VTE Risk Stratification in MHT

Evaluation of venous thromboembolism risk is central to the benefit–risk assessment of menopausal hormone therapy. Most data concern oral estrogen, alone or combined with a progestogen, and consistently indicate that VTE risk varies by route of administration, formulation, and dose.

Large, population-based analyses show that transdermal estradiol—used either alone or with a progestogen—is not associated with an increased VTE risk, whereas oral estrogen regimens are linked to a higher risk [45]. A major UK case–control study assessing commonly used preparations further reported that oral conjugated equine estrogen, whether estrogen-only or combined, is associated with a higher VTE risk than oral estradiol; a clear dose–response relationship was observed (higher estrogen dose corresponding to greater VTE risk). In the same dataset, transdermal MHT and tibolone—used less frequently—were not associated with excess VTE, findings aligned with earlier observational studies [47,48,49,50].

These findings were corroborated by a recent large U.S. cohort, which examined over 20,000 VTE cases and confirmed that oral estradiol carries a lower thrombotic risk than CEEs, while transdermal estradiol remains risk-neutral [51]. In the same cohort, combined contraceptive exposures with ethinyl estradiol showed substantially higher VTE risk than menopausal regimens, and concurrent statin therapy appeared to attenuate MHT-related VTE risk [52].

Complementing these results, a recent systematic review focusing on women with additional VTE risk factors concluded that transdermal MHT appears to have the most favorable safety profile, oral estrogen confers a higher risk, and oral estrogen–progestogen combinations confer the highest risk [53].

Among oral preparations, CEEs confer a higher risk of venous thromboembolism than oral estradiol, consistent with observational and case–control studies. The choice of progestogen further modulates risk, with micronized progesterone and dydrogesterone showing the most favorable profiles.

In clinical practice, symptomatic women with elevated baseline VTE risk (for example, obesity, major immobility, strong family history or selected thrombophilias) should preferentially receive transdermal estradiol, with the use of the lowest effective systemic dose; oral regimens should be reserved for carefully selected cases with appropriate counseling regarding dose-dependent risk.

#### 2.3.3. Breast Cancer Outcomes with MHT

The association between menopausal hormone therapy and breast cancer remains debated, with observational and randomized data yielding partly divergent estimates. In long-term follow-up of the two WHI randomized trials, estrogen alone in women with prior hysterectomy was associated with lower breast cancer incidence and mortality, whereas estrogen–progestin (CEE+MPA) in women with an intact uterus was associated with higher incidence and no clear difference in mortality [54].

Large population-based analyses from the UK refine risk by formulation, duration, and patient factors. Excess risk is predominantly linked to estrogen–progestogen regimens, with smaller increases observed for estrogen-only therapy; risks rise with longer duration and decline after cessation. Among combined regimens, estradiol–dydrogesterone showed the lowest associated increase, whereas longer exposures to medroxyprogesterone acetate, norethisterone, and levonorgestrel carried higher risks. Associations tended to be more pronounced at older ages and attenuated in obesity [55].

In breast cancer survivors, a recent systematic review and meta-analysis found that systemic MHT increased recurrence risk versus placebo, driven largely by hormone-receptor-positive disease and not observed in receptor-negative tumors [56].

Contemporary mechanistic and clinical syntheses suggest that progestogens are the principal hormonal drivers of MHT-related breast cancer risk, with estrogens potentially contributing indirectly via progesterone receptor induction and amplification of progesterone signaling. Consistent with this, combined estrogen–progestin therapy increases risk, whereas estrogen-only MHT shows little or no increase and, in extended WHI follow-up, was associated with lower incidence and mortality. Transient high-estradiol states (for example, during ART) appear neutral for risk or recurrence; the efficacy of antiestrogens likely reflects suppression of progesterone signaling [57].

An updated British Menopause Society consensus synthesizing recent meta-analyses and WHI data concluded that, in women at low baseline risk, a five-year course of MHT for symptom control generally outweighs potential harms. Estrogen-only therapy is not associated—or only minimally associated—with increased risk; low-dose local vaginal estrogen is not associated with increased risk; and combined MHT is associated with a duration-dependent increase [58].

Differences between continuous and sequential combined regimens are small and may be subordinated to endometrial protection considerations. The statement advises avoiding certain synthetic progestogens to minimize risk, notes that risk is more evident in normal-weight than in obese women, and clarifies that in premature ovarian insufficiency, years of exposure are counted from age 50. For high-risk women (for example, strong family history or high-risk benign disease), no clear additive risk of MHT has been demonstrated; local vaginal estrogen may be used with tamoxifen but generally not alongside aromatase inhibitors [58].

#### 2.3.4. MHT and Ovarian Cancer Risk

Since the WHI era, multiple investigations have re-examined ovarian risk. A meta-analysis of 52 epidemiological studies (since 1970) estimated that initiating MHT for about five years around age 50 yields approximately one additional ovarian cancer per 1000 users; risk declines after cessation but remains appreciable in the first few years and may persist at a low level even a decade later [59]. Among recent users (initiated within the past five years), the relative risk did not differ materially between estrogen-only and estrogen–progestogen regimens or by age at initiation (<50 versus during the 50s). Risk varied by histologic subtype—increased for serous and endometrioid tumors, with a possible decrease for clear-cell tumors [59].

EMAS advises caution with MHT in women with serous epithelial ovarian cancer, while NAMS (level II) notes a small but statistically significant ovarian cancer risk with hormone use in observational studies [60].

In a 2024 systematic review and meta-analysis, MHT use was associated with a small increase in ovarian cancer risk, but this signal attenuated in more recent studies (no significant excess in cohorts after 2010 or case–control studies after 2006). Risk rose with longer duration—particularly >10 years—and appeared higher for serous histology. Estrogen-only therapy may contribute to the excess with prolonged use, whereas combined estrogen–progestogen regimens, including continuous schedules, showed risks comparable to sequential use. Overall, the authors conclude that ovarian cancer risk associated with MHT has decreased over time; for long-term users, continuous estrogen–progestogen regimens may represent a safer alternative to extended estrogen-only therapy [61].

#### 2.3.5. Comorbidities Relevant to MHT Use: Biliary Disease and Hypertension

Observational and trial data consistently indicate that oral estrogen increases the risk of symptomatic gallstone disease and cholecystectomy, with risk rising with duration of use. Transdermal estradiol, which avoids first-pass hepatic metabolism, appears to confer a lower hepatobiliary burden. In women with active or prior gallbladder disease, a transdermal route is generally preferred, using the lowest effective dose and periodic reassessment [62,63].

Compared with transdermal or local vaginal preparations, oral estrogen has been associated with higher rates of incident hypertension and less favorable blood pressure trajectories. When treating women with established hypertension or elevated baseline cardiovascular risk, transdermal estradiol is usually favored; dose titration should target symptom control while avoiding upward pressure on blood pressure [64].

### 2.4. Emerging Therapies: Estetrol (E4)

Among the four naturally occurring human estrogens (estrone, estradiol, estriol, and estetrol), important pharmacologic and metabolic distinctions exist. Estradiol is the most potent and commonly used form in menopausal hormone therapy, whereas estrone predominates in postmenopausal circulation and has weaker systemic effects. Estriol exhibits primarily local urogenital activity and is used in low-dose vaginal applications. By contrast, estetrol is produced only during fetal life, displays much higher oral bioavailability and slower elimination than E2, is not further metabolized into more active estrogenic metabolites, and has minimal hepatic activation and influence on hemostatic and drug-metabolizing enzymes—features that suggest a potentially safer vascular and thrombotic profile [65].

Estetrol is a naturally occurring fetal estrogen with distinct pharmacology that has entered clinical use in combined oral contraception, with drospirenone, and is under evaluation for menopausal hormone therapy. E4 displays estrogenic activity in uterovaginal tissues, bone, cardiovascular and central nervous systems, with comparatively limited hepatic impact (including on hemostatic parameters). Unlike estradiol, E4 has high oral bioavailability, metabolic stability, and undergoes predominantly phase-II conjugation; cytochrome P450 enzymes make only a minor contribution to its metabolism, and E4 is not back-converted to other active estrogens (E1/E2/E3), functioning as a terminal metabolite [65].

In the randomized dose-finding phase II trial E4Relief (NCT02834312), postmenopausal women with moderate–severe vasomotor symptoms received E4 at 2.5, 5, 10, or 15 mg daily for 12 weeks. A clinically and statistically significant reduction in hot-flash frequency/severity was demonstrated at 15 mg, which was selected as the minimal effective daily dose for VMS treatment [66]. Endometrial effects were dose-related increases in thickness during treatment without cases of hyperplasia; thickness returned to baseline after a 14-day course of dydrogesterone 10 mg at study end, indicating the need for endometrial protection when the uterus is present [66].

In the same study, E4 improved vaginal maturation indices and reduced signs of genital atrophy; vaginal pH decreased across E4 arms (versus a slight rise on placebo), and patient-reported vaginal dryness (15 mg) and pain (5–15 mg) improved significantly, whereas irritation/itching and dysuria did not [66].

Skeletal biomarkers in E4Relief showed a decrease in CTX-1 from baseline in the 5–15 mg groups, with statistically significant reductions versus placebo at 10 and 15 mg; osteocalcin changes from baseline were not significant within groups but were significant versus placebo after 12 weeks. These findings support a potential antiresorptive signal that warrants longer-term evaluation with bone density and fracture outcomes [67].

Preclinical studies suggest favorable vascular actions (nitric oxide production, vasodilation, endothelial repair, anti-atherosclerotic effects), and E4′s effect on endothelial migration was comparable to ethinylestradiol; however, clinical cardiovascular outcome data in postmenopausal women are not yet available [68].

Breast data remain limited but reassuring in early studies: in clinical samples, E4 exhibited a pro-apoptotic effect in tumor tissue without changes in Ki-67 proliferation indices in pre- and postmenopausal women [65]. Metabolic readouts in E4Relief showed minimal changes in triglycerides versus placebo, HDL-C increased across E4 groups, and small increases in LDL-c/total cholesterol at lower doses that were not significant versus placebo; fasting glucose was unchanged, while insulin resistance and HbA1c decreased significantly at 10 mg and 15 mg, respectively—suggesting improved glucose tolerance [65,67].

Venous thromboembolism risk with postmenopausal E4 has not yet been characterized in dedicated outcome studies. Signals from contraceptive settings indicate a potentially lower thrombotic profile for E4-drospirenone compared with ethinylestradiol-based combined pills, with the lowest disproportionality reporting for thrombotic events observed for E4-drospirenone, approaching that of progestin-only pills [65,69,70]. Extrapolation to MHT requires caution until postmenopausal VTE data are available.

### 2.5. Practice Synthesis—Society Guidance at a Glance

Updated recommendations from key MHT guidelines are summarized in a table to streamline clinical application (Table 3).

## 3. Conclusions

Menopausal hormone therapy remains the most effective intervention for vasomotor symptoms and a cornerstone for genitourinary syndrome of menopause, with additional benefits for early postmenopausal bone health and patient-reported outcomes. Across outcomes, effect size and safety are contingent on timing, route, dose, and progestogen: initiation within a decade of menopause favors benefit–risk; transdermal estradiol at low–moderate doses are preferred when cardiometabolic or thrombotic risk is salient; and endometrial protection is mandatory in women with a uterus.

Randomized and population data converge that MHT is not indicated for primary cardiovascular prevention; oral regimens are associated with higher risks of venous thromboembolism and, in aggregate, stroke. Breast cancer associations are regimen-specific: neutral to favorable with estrogen-alone after hysterectomy; and duration-dependent excess with combined therapy. Ovarian cancer signals are small in absolute terms and require individualized counseling. Evidence for colorectal cancer remains mixed and should be interpreted cautiously.

Emerging options may refine tolerability. Estetrol demonstrates anti-VMS efficacy at the minimum effective oral dose, limited hepatic impact, and early signals for vaginal health and bone turnover; however, definitive cardiovascular, thrombotic, and long-term breast outcomes are pending, and progestogen co-administration remains necessary where indicated.

In practice, MHT should be individualized through shared decision-making—the right patient, timing, route, dose, and progestogen—using the lowest effective dose with periodic re-evaluation. Continued head-to-head trials of routes and progestogens and robust long-term safety data for E4 are priorities to sharpen risk stratification and widen therapeutic choice.

## Figures and Tables

**Table 1:** Estrogen therapy formulations/routes: advantages, adverse effects, and practical notes.

Route	Examples/Formulations	Advantages	Adverse Effects/Limitations	Notes/When to Prefer
Oral (systemic)	17β-estradiol tablets (e.g., 0.5–2 mg/day); conjugated equine estrogens (CEEs 0.3–0.625 mg/day); synthetic conjugated estrogens	Convenient-generally reliable absorption	Nausea, breast tenderness, headache; ↑ triglycerides; ↑ thyroid-binding globulin (may require LT4 dose adjustment); ↑ risk of VTE and gallbladder disease [5]	Avoid in hypertriglyceridemia or elevated VTE risk; consider in women who prefer pills and have low vascular risk; progestogen needed if uterus present
Transdermal (systemic)	Estradiol patch (25–100 μg/day), gel, or spray	Bypasses first-pass hepatic metabolism; minimal effect on hepatic coagulation proteins and TBG at standard doses; lower impact on VTE risk; lipid-neutral profile [6]	Local skin irritation (less with gels); rare allergy; adherence issues (patch changes); occasional suboptimal absorption [8]	Often preferred in higher VTE risk, migraine with aura, smokers ≥ 35, hypertension, or hypertriglyceridemia; flexible dosing; progestogen needed if uterus present
Vaginal (low-dose, local)	Estradiol 10 μg tablets; estradiol ring (e.g., 7.5 μg/day); low-dose creams; vaginal DHEA 6.5 mg	Highly effective for GSM (vaginal dryness, dyspareunia, recurrent UTIs); minimal systemic absorption; typically, no progestogen required at low doses [10]	Local irritation/discharge; does not reliably treat systemic VMSs; cost considerations [10]	Use when symptoms are genitourinary-predominant or systemic MHT is contraindicated; can be combined with systemic MHT if needed

Abbreviations: VTE = venous thromboembolism; TBG = thyroid-binding globulin; GSM = genitourinary syndrome of menopause; LT4 = levothyroxine; VMSs = vasomotor symptoms, ↑ = elevated.

**Table 2:** Typical systemic estrogen dosing.

Dose Category	Oral Conjugated Estrogens	Oral Micronized 17β-Estradiol (E2)	Transdermal E2—Patch (Delivery Rate)
Ultra-low	0.3 mg/day	0.25 mg/day	0.014 mg/day (14 μg/day)
Low	0.45 mg/day	0.5 mg/day	0.025 mg/day (25 μg/day)
Standard	0.625 mg/day	1 mg/day	0.0375–0.05 mg/day (37.5–50 μg/day)

**Table 3:** Summary of international guidelines on menopausal hormone therapy.

Society/Guideline	Year/Latest Update	Main Indications	Key Recommendations	Warnings/Contraindications
NAMS (The Menopause Society) [6]	2022–2024	Moderate–severe VMSs, GSM, prevention of bone loss and fractures	Initiation < 60 years or <10 years since menopause → best benefit–risk ratio (‘timing hypothesis’) MHT is the most effective treatment for VMS and GSM Prefer transdermal route in women at increased VTE risk Progestogen required if uterus present Continuation > 65 years may be acceptable in selected women	Periodic benefit–risk re-assessment Avoid oral estrogen in active/recent VTE, stroke or breast cancer
EMAS (European Menopause and Andropause Society) [71]	2024–2025 (topic-based updates)	Natural or surgical menopause, special situations (endometriosis, POI)	Strong emphasis on individualization by age, time since menopause, and risk profile Prefer transdermal estrogen in women at increased VTE risk In endometriosis: continuous combined MHT even after hysterectomy For POI: MHT recommended until the average age of natural menopause	Avoid estrogen-only therapy in endometriosis Regular clinical and imaging follow-up
ACOG (American College of Obstetricians and Gynecologists)	2023 (Practice Bulletin & Committee Opinions)	VMS, GSM, bone loss prevention in symptomatic women	Use lowest effective dose for shortest necessary duration Individualize therapy; annual review Add progestogen if uterus present Compounded ‘bioidentical’ MHT not recommended—prefer FDA-approved formulations	Avoid in active breast cancer, VTE, severe liver disease, unexplained bleeding Caution in high cardiovascular risk
BMS (British Menopause Society) [37]	2024–2025	VMS, GSM, osteoporosis prevention	MHT = first-line option for menopausal symptoms (aligned with NICE NG23) Provides detailed dose equivalence charts and titration guidance Ensure adequate endometrial protection proportional to estrogen dose Prefer micronized progesterone or LNG-IUS	Discuss absolute risks (breast cancer, VTE) during counseling Avoid late initiation (>60 years) unless clinically justified
Endocrine Society [72]	2015 (still current)	Moderate–severe VMSs, osteoporosis prevention	Individualize route and dose Add progestogen if uterus present Best benefit–risk if started <60 years/<10 years post-menopause Shared decision-making encouraged	Contraindicated in active VTE, breast cancer, severe liver disease Reassess every 6–12 months

## Data Availability

No new data were created or analyzed in this study. Data sharing is not applicable to this article.

## Abbreviations

The following abbreviations are used in this manuscript:

NHIS-NSC	National Health Insurance Service–National Sample Cohort (Korea)
ASCVD	Atherosclerotic Cardiovascular Disease
ELITE	Early Versus Late Intervention Trial With Estradiol
EMAS	European Menopause and Andropause Society
KEEPS	Kronos Early Estrogen Prevention Study
E4Relief	E4Relief Phase II Trial
CTX-1	C-terminal Telopeptide Of Type I Collagen
NAMS	North American Menopause Society
NK3R	Neurokinin-3 Receptor
DHEA	Dehydroepiandrosterone
SHBG	Sex Hormone-Binding Globulin
HDL-c	High-Density Lipoprotein Cholesterol
LDL-c	Low-Density Lipoprotein Cholesterol
SNRI	Serotonin–Norepinephrine Reuptake Inhibitor
SSRI	Selective Serotonin Reuptake Inhibitor
ART	Assisted Reproductive Technology
BMS	British Menopause Society
CEEs	Conjugated Equine Estrogens
CHD	Coronary Heart Disease
CRC	Colorectal Cancer
CVD	Cardiovascular Disease
EPT	Estrogen–Progestogen Therapy
ERα	Estrogen Receptor Alpha
ERβ	Estrogen Receptor Beta
FMD	Flow-Mediated Dilation
GSM	Genitourinary Syndrome of Menopause
MHT	Menopausal Hormone Therapy
MPA	Medroxyprogesterone Acetate
NMD	Nitroglycerin-Mediated Dilation
TBG	Thyroxine-Binding Globulin
UTIs	Urinary Tract Infections
VMSs	Vasomotor Symptoms
VTE	Venous Thromboembolism
WHI	Women’s Health Initiative
LT4	Levothyroxine
E2	17β-Estradiol
E4	Estetrol
ET	Estrogen Therapy
PR	Progesterone Receptor
